# Complete Genome Sequence of *Bacillus velezensis* CBMB205, a Phosphate-Solubilizing Bacterium Isolated from the Rhizoplane of Rice in the Republic of Korea

**DOI:** 10.1128/genomeA.00654-16

**Published:** 2016-07-14

**Authors:** Kyeong Hwangbo, Yurry Um, Ki Yoon Kim, Munusamy Madhaiyan, Tong Min Sa, Yi Lee

**Affiliations:** a Department of Industrial Plant Science and Technology, Chungbuk National University, Cheongju, Republic of Korea; b Department of Herbal Crop Research, National Institute of Horticultural and Herbal Science, Rural Development Administration, Eumseong, Republic of Korea; c Department of Environmental and Biological Chemistry, Chungbuk National University, Cheongju, Republic of Korea; d Biomaterials and Biocatalysts Group, National University of Singapore, Singapore

## Abstract

*Bacillus velezensis* CBMB205 (= KACC 13105^T^ = NCCB 100236^T^) was isolated from the rhizoplane of rice (*Oryza sativa* L. cv. O-dae). According to previous studies, this bacterium has several genes that can promote plant growth, such as the phosphorus-solubilizing protein-coding gene. Here, we present the first complete genome of *B. velezensis* CBMB205.

## GENOME ANNOUNCEMENT

*Bacillus velezensis* (synonym, *Bacillus methylotrophicus* [1]) strain CBMB205 is a novel bacterium isolated from the rhizoplane of rice (*Oryza sativa* L. cv. O-dae), from samples collected by the Chungbuk Provincial Agricultural Research and Extension Services (Cheongwon, Republic of Korea) in 2010. This type strain bacterium has the methylotrophic ability to use C1 compounds, such as methanol, as an energy source. The bacterium is characterized as Gram-positive, aerobic, motile, rod-shaped, and forming endospores (2). According to 16s rRNA sequencing analysis, the bacterium is a new member of the genus *Bacillus*. Genomic DNA of CBMB205 was purified using the PowerSoil DNA isolation kit, and before sequencing with the PacBio RS II system (3), we constructed a 20-kb library following the manufacturer’s instructions. The reads were *de novo* assembled using PacBio SMRT Analysis version 2.3.0, and comparisons of the predicted open reading frames using the SEED (4), Clusters of Orthologous Groups (5), EzTaxon-e (6), and Pfam databases were conducted during gene annotation (7). Additional gene prediction analysis and functional annotation were performed with the RAST server (8) and GLIMMER version 3.02 (9). RNAmer version 1.2 (10) and tRNAscan-SE version 1.23 (11) were used to identify rRNA genes and tRNA genes, respectively.

In all, 77,620 reads with 259.52× coverage were produced. The genome of *B. velezensis* CBMB205 has a 3,929,745-bp circular chromosome with a GC content of 46.50%, 27 rRNAs, and 86 tRNAs. CBMB205 has 4,113 genes and a total of 3,985 coding sequences (CDSs), including 2,836 function-assigned CDSs.

The bacterium has genes that can help plant growth-promoting functions. For example, CBMB205 has 19 phosphatase genes (including a single phytase gene; CBMB205_20350). These genes are involved in solubilizing phosphorus to mineral phosphorus that can be used by plants. Phosphorus is known as one of the essential elements for plant growth (12). Moreover, the *B. velezensis* CBMB205 genome has 14 thiamine synthesis-related genes. Thiamine is known as an essential cofactor required for carbohydrate and amino acid metabolism in bacteria. Also, the formation of vitamin B by *Bacillus* and its growth-promoting effects on plants have already been described (13), and several experiments have revealed that CBMB205 has catalase, oxidase, pectinase, and protease activities.

This study presents the first fully sequenced and annotated strain of *B. velezensis* CBMB205, which is valuable for studying its physiology, evolution, ecology, and biotechnological applications.

### Nucleotide sequence accession number.

The complete chromosome sequence has been deposited at DDBJ/EMBL/GenBank under accession number CP014838. The version described in this paper is the first version.
